# Supplementary material for: Automatic calibration of a multi-camera system with limited overlapping fields of view for 3D surgical scene reconstruction
Source: Int J Comput Assist Radiol Surg. 2025 Jun 30;21(1):175–83. doi: 10.1007/s11548-025-03413-1 (PMC12929363; doi:10.1007/s11548-025-03413-1)
Supplement: Supplementary file 1 — (pdf 3116 KB) [file 11548_2025_3413_MOESM1_ESM.pdf]

Online Resource 1

Automatic Calibration of a Multi-Camera System with Limited Overlapping Fields of View for 3D Surgical Scene Reconstruction

International Journal of Computer Assisted Radiology and Surgery (IJCARS) - IPCAI 2025 Special Issue

Tim Flückiger[1,2*], Jonas Hein[1,2], Valery Fischer[1], Philipp Fürnstahl[1], Lilian Calvet[1]

[1]Research in Orthopedic Computer Science, University Hospital Balgrist, University of Zurich, Switzerland.
[2]Computer Vision and Geometry, ETH Zurich, Switzerland.

*Corresponding author(s). E-mail(s): tflueckiger@ethz.ch;

This supplementary material provides technicalities and additional results of the experiments that were omitted in the main paper due to space limitations. We provide the reader results of a preliminary experiment on marker systems, additional implementation details in Section 2.2, and present additional quantitative results in Section 3.

1 Preliminary experiment

As explained in the main paper, planar patterns and their corresponding detection functions used in practice to create multi-scale markers (MSMs) are taken from existing state-of-the-art fiducial marker systems. Before evaluating the complete system, we conducted experiments on synthetic images to quantify the detection rates and localization errors of state-of-the-art marker systems. This property is critical in our application for achieving a fast and accurate multi-camera calibration. A higher detection rate across a wide range of marker sizes allows for reduced projection times,

as fewer scales need to be projected. The experiments were carried out on synthetic images generated using the Blender software.

We evaluated three marker systems: ArUco [1], AprilTag [2], and CCTag [3]. ArUco and AprilTag are square fiducial marker systems, and widely used for pose estimation, camera calibration, and object tracking. For each system, two dictionaries were evaluated to cover a broader range of scenarios. Additionally, we evaluated CCTag, a circular fiducial marker system designed for robust detection under challenging conditions such as extreme motion blur and lighting conditions.

Scene The synthetic scene consists of a single marker of varying sizes projected onto a floor plane. The marker is placed at the origin of the scene in the xy-plane. We placed 15 cameras at various sample locations within the volume likely to be covered during 3D-SSR (see Fig. 1). The distance ranges from 1.8 to 6.7 meters, and the viewing angle ranges from 16° to 90°. The scene was tested under three different lighting conditions by modifying the alpha blending between the projected pattern and the floor texture. To ensure a fair comparison between the three marker systems, we ensure that all three marker types cover the same area at a given scale.

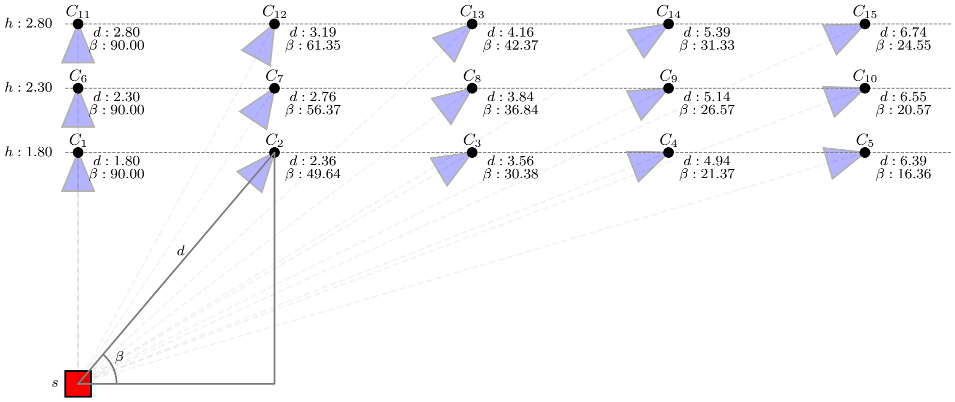

Figure 1 Cameras placement for the evaluation of marker systems

Camera The cameras follow a pinhole camera model with square pixels. The principal point is located at the center of the image, with a focal length of 1040 pixels and an image resolution of 1920x1080 pixels (Full HD).

Image Corruption In addition to simulating different lighting conditions, we introduced image corruption via Gaussian blur and noise. Gaussian blur was applied using kernel sizes of 0, 3, or 5 pixels to simulate depth-of-field blur, which could arise from both the marker projection and the cameras. We also added additive white Gaussian noise with zero mean and standard deviations of 0, 5, or 10 pixels to simulate varying levels of sensor noise.

Results Fig. 2a shows the detection rate as a function of the marker size. For each marker size, 405 images (15 cameras, 3 lighting conditions, 3 Gaussian blur kernel sizes, and 3 noise levels) are created to evaluate the detection rate. AprilTag presents the highest overall detection rate. For completeness, we tested two different dictionnaries

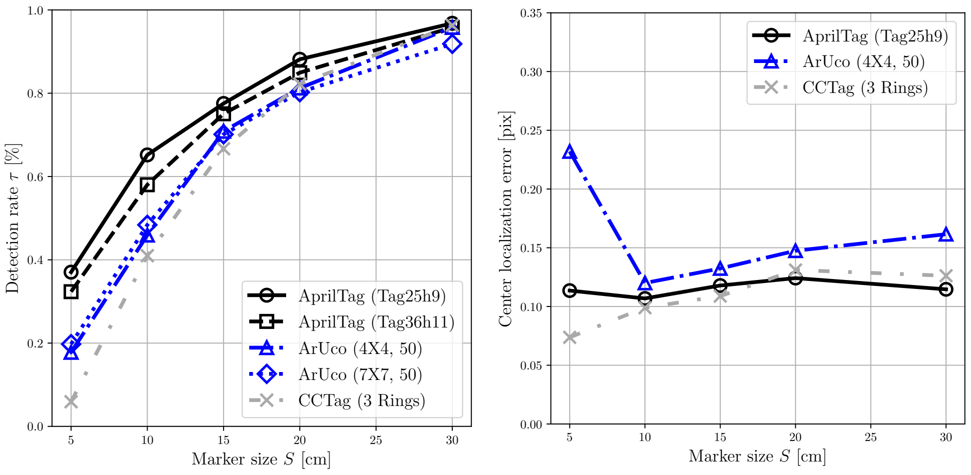

(a) (b)

Figure 2 (a) Detection rate vs. the marker size S. (b) Center localization error vs. the marker size S

for AprilTag and ArUco. The one using more encoding bits (AprilTag Tag36h11, ArUco 7X7) have lower detection rates. This result is expected since they are more difficult to detect due to the higher number of bits that need to be extracted from the image. Fig. 2b presents the localization errors for both the corners and the centers of the different marker systems. All tested systems achieve subpixel accuracy. Although ArUco performs slightly worse on synthetic data, it shows slightly better results on real data (in terms of reprojection error), which motivated us to report the results obtained using MSMs made of ArUco markers in the main paper.

2 Implementation details

2.1 Different levels of constraint

We recall that the external calibration problem is that of finding a set of camera poses that best explain the extracted imaged MSMs centers. It is stated as a non-linear least squares optimization problem, commonly known as bundle adjustment (BA), whose solution is

$$\arg\min_{\{\mathbf{P}_c\},\{\mathbf{X}_k\}} \sum_c \sum_k v_{c,k} \left\| proj\left(\mathbf{K}_c, \mathbf{P}_c, \mathbf{X}_k\right) - \mathbf{x}_{c,k} \right\|_2^2 \tag{1}$$

where $\mathbf{P}_c$ are the camera poses parameters (parametrizing the rotation and translation), $\mathbf{K}_c$ the (known) camera intrinsics, $\mathbf{X}_k$ the center of the MSM k, $\mathbf{x}_{c,k}$ its image in the camera c, $v_{c,k}$ equal to 1 if $\mathbf{X}_k$ is observed in camera c, 0 otherwise, and $proj$ the projection function of the camera model.

This formulation in Eq. 1 is free of constraints on the 3D points, but it can easily integrate constraints. We consider three cases:

1. *free*: no constraint (Eq. 1)
2. *coplanarity*: points are on a plane π (Eq. 2)
3. *homography*: coplanarity constraint and enforcing that the 2D coordinates of the points in the plane π and their coordinates in the projector image are linked by an homography $\mathbf{H}^{\pi}_{proj}$, (Eq. 3)

For the *coplanarity* case, the problem becomes:

$$\arg\min_{\{\mathbf{P}_c\},\{\mathbf{x}^{\pi}_k\}} \sum_c \sum_k v_{c,k} \left\| proj\left(\mathbf{K}_c, \mathbf{P}_c, \mathbf{x}^{\pi}_k, \mathbf{P}_{\pi}\right) - \mathbf{x}_{c,k} \right\|_2^2 \tag{2}$$

with $\mathbf{x}^{\pi}_k$ the 2D coordinates of the points in the plane π and $\mathbf{P}_{\pi}$ the pose of the plane π.

If, in addition, we enforce that the 2D coordinates of the points in the plane π and their coordinates in the projector image are linked by an homography $\mathbf{H}^{\pi}_{proj}$ (*homography* case), the points are fully determined by the plane pose $\mathbf{P}_{\pi}$ and the homography $\mathbf{H}^{\pi}_{proj}$:

$$\arg\min_{\{\mathbf{P}_c\},\{\mathbf{x}^{\pi}_k\}} \sum_c \sum_k v_{c,k} \left\| proj\left(\mathbf{K}_c, \mathbf{P}_c, \mathbf{P}_{\pi}, \mathbf{H}^{\pi}_{proj}\right) - \mathbf{x}_{c,k} \right\|_2^2 \tag{3}$$

In this case the homography $\mathbf{H}^{\pi}_{proj}$ is parametrized via the 4-point parametrization [4]. This assumes that the projector does not have any distortion in the projection (i.e. that is satisfies a pinhole camera model). To satisfy with this assumption, the projector can be calibrated [5], and images can be distorted with the calibrated coefficients before projection to compensate for the lens distortion. In this case, there is effectively a homography linking the 2D points in the plane and their coordinates in the original image before distortion. This link enables for the calibration of cameras with no overlap. This is possible since once an estimate of the pose of π and $\mathbf{H}^{\pi}_{proj}$ is

known, the 3D coordinates of all MSM centers can immediately be inferred, thus providing 2D-3D correspondences with any camera seeing the projection surface (even if it has strictly no overlap fov with any other camera), allowing for retrieving its pose using the PnP algorithm. In the case where no camera has any overlap with any other, the relative motion of the first camera pair can still be retrieved. Indeed, even though the inter-image homography cannot be estimated directly from a set of inter-view correspondences, we can use homography composition. First, inter-image homography H_{Ci}^{proj} is computed between each of the two cameras and the projector. Then, the inter-image homography of the camera pair can be estimated as the composition of these homographies $H_{C1}^{C2} = (H_{C2}^{proj})^{-1} H_{C1}^{proj}$.

2.2 Calibration algorithm

We provide the detailed algorithm presented in the main paper for the *free* case as the two other cases follow the same structure.

Algorithm 1 External calibration of a multi-camera system using weakly coplanar points.

Require: A set of 2D correspondences in cameras indexed $1, ..., n$
Ensure: Camera poses $\{\mathbf{P}_i\}$ and 3D points $\mathcal{X}$.

$\mathcal{C} \leftarrow \{\}$, $\mathcal{X} \leftarrow \{\}$
Select an initial camera pair (i_1, i_2) maximizing the view scores $s(i_1)$ and $s(i_2)$.
Estimate the inter-image homography $H_{i_2}^{i_2}$ between cameras i_1 and i_2.
Retrieve the pose $\mathbf{P}_{i_2}$ of camera i_2 w.r.t. camera i_1 by decomposing $H_{i_2}^{i_2}$.
$\mathcal{C} \leftarrow \mathcal{C} \cup \{i_1, i_2\}$
Triangulate 3D points $\{\mathbf{X}_k\}$ from the initial camera pair (i_1, i_2).
$\mathcal{X} \leftarrow \mathcal{X} \cup \{\mathbf{X}_k\}$
while $|\{1, ..., n\} \setminus \mathcal{C}| > 0$ **do**
    Select camera $j \in \{1, ..., n\} \setminus \mathcal{C}$ with the highest view score $s(j)$ based on the correspondences with the triangulated points $\mathcal{X}$.
    Estimate pose $\mathbf{P}_j$ of camera j using 2D-3D correspondences via PnP algorithm.
    Triangulate new 3D points using each new camera pair (j, i) with $i \in \mathcal{C}$.
    $\mathcal{C} \leftarrow \mathcal{C} \cup \{j\}$
    $n_{\text{out}} \leftarrow +\infty$
    while $n_{\text{out}} > 0$ **do** ▷ Perform iterative BA and outlier filtering
        Solve intermediate BA (Eq. 1) over estimated cameras $\mathcal{C}$ and 3D points $\mathcal{X}$.
        Filter and count the number of observations n_{out} with a reprojection error higher than a threshold ϵ.
    end while
end while

3 Additional Results

In this section we provide additional results obtained in the real-world experiment. A detailed top-down view of each of the four camera setups (*full*, *easy*, *medium*, *hard*) is shown in Fig. 3. Table 1 and Table 2 present the results of the same experiment on real data as reported in the main paper, this time using MSMs composed of AprilTag markers. The performance with AprilTags is slightly lower than with ArUco markers. Additionally, as shown in Table 2, there is no significant difference between results with or without the coplanarity constraint integrated into the BA (difference smaller than 0.01 pixel). Fig. 4 shows the detected MSM centers in the camera images along with their corresponding reprojection errors. Similarly, Fig. 5 presents the detected points from the evaluation data and their corresponding reprojection errors.

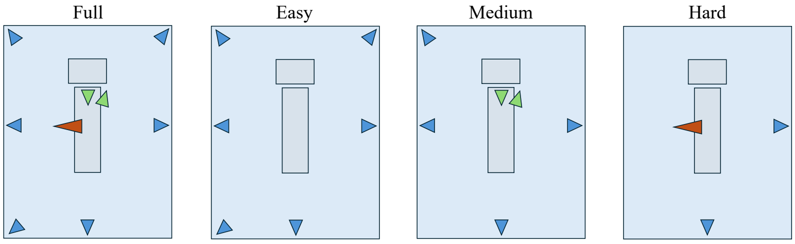

Figure 3 Top-down view of the selected cameras configurations (*Full*, *Easy*, *Medium*, *Hard*). Blue: far-field (GoPros), green: near-field cameras (GoPros), orange: close-up camera (Canon with strong optical zoom). One of the near-field camera is oriented towards the instrument table

	Full (6/2/1)		Easy (6/0/0)		Medium (4/2/0)		Hard (2/0/1)	
	Free	Copl.	Free	Copl.	Free	Copl.	Free	Copl.
Calibration	0.28	0.35	0.27	0.34	0.25	0.32	0.12	0.23
Evaluation	0.33	0.33	0.3	0.29	0.34	0.34	0.3	0.27

Table 1 Reprojection errors on calibration and evaluation data for the proposed calibration method on distinct setups, in the *free* (Eq. 1) and *coplanarity* case integrating the coplanarity constraint in the optimization (Eq. 2), using MSMs made of AprilTag markers

		Far Field (6)		Near Field (2)		Close-up (1)		All (9)	
		Free	Copl.	Free	Copl.	Free	Copl.	Free	Copl.
	Repr. err.	0.29	0.35	0.22	0.30	0.05	0.06	0.28	0.35
Calib.	Distrib. score	208	208	316	316	376	376	251	251
	# obsv.	3026	3011	1315	1301	49	49	2315	2302
Eval.	Repr. err.	0.36	0.35	0.20	0.19	0.06	0.07	0.33	0.33

Table 2 Results per camera category on the *full* setup for the proposed method, for the *free* (Eq. 1) and *coplanarity* case (Eq. 2), using MSMs made of AprilTag markers. Mean reprojection error (calib. & eval.), disribution score (calib., see main paper subsection 2.2), observations of 3D points with track length >= 2 (calib.)

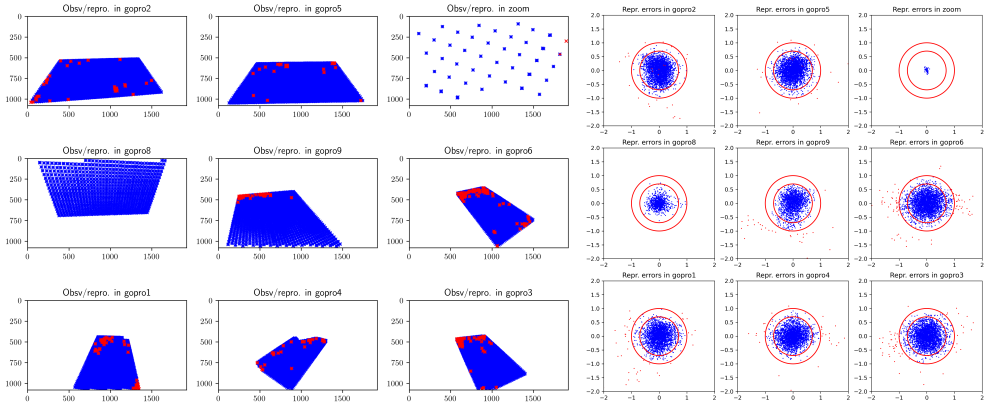

(a) (b)

Figure 4 Calibration data for the proposed method on the *full* setup, in the *coplanarity* case, using MSMs made of ArUco markers : (a) observations and reprojections, (b) reprojection errors

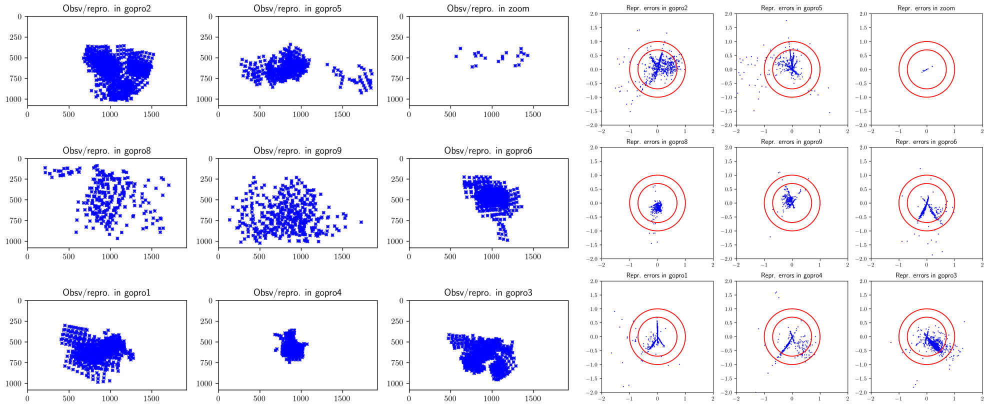

(a) (b)

Figure 5 Evaluation data for the proposed method on the *full* setup, in the *coplanarity* case, using MSMs made of ArUco markers : (a) observations and reprojections, (b) reprojection errors

We provide an additional qualitative analysis of an extreme case involving only two cameras (one positioned in the far field and the other in the near field). This experiment compares the ChArUco method against our proposed approach and serves as an ablation study contrasting a fixed-size marker (baseline) with a collection of markers of varying sizes (proposed MSM). It is conducted using a camera-pair, taken in isolation, out of the camera setup used in the real experiment described in the main article. Specifically, a ChArUco board of fixed size—chosen as the optimal trade-off for detection in both far-field and near-field cameras—is positioned to ensure that it is seen by the near-field camera. We analyze the number of correspondences provided by the fixed-size marker compared to the proposed collection of varying-sized markers (MSM). In the case of the ChArUco board, no correspondences are found because the corners are not detected by the far-field camera while only 2 are detected in the near field camera. In contrast, with the proposed MSM, 41 inter-viewpoint correspondences are identified (indicated by red crosses in the bottom row of Fig. 6).

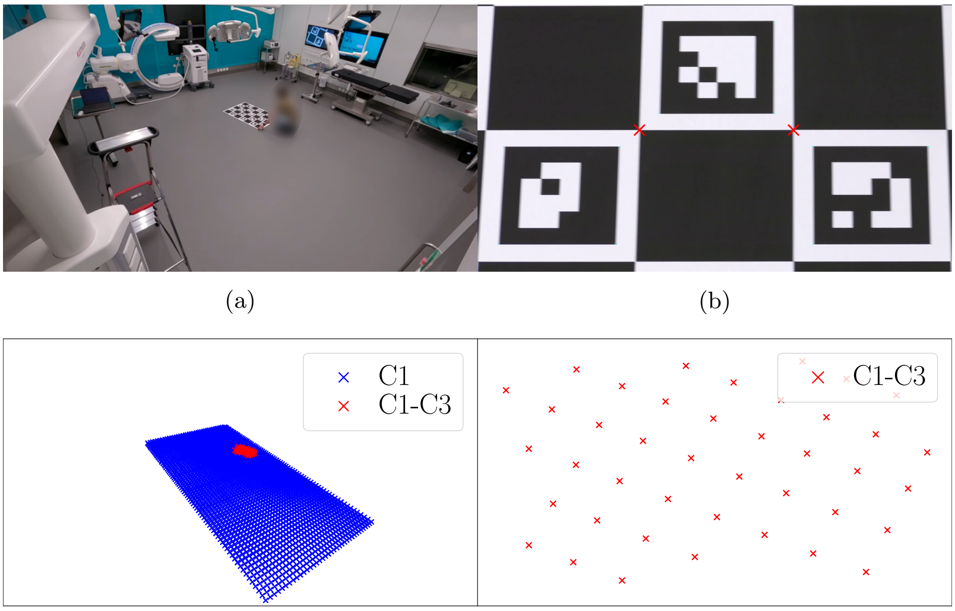

(a) (b)

(c) (d)

Figure 6 Example of a setup with two cameras: one positioned in the far field and the other ceiling-mounted with strong optical zoom. Top: The ChArUco board of fixed size shows (a) no detected corners in the far-field camera and (b) only two detected corners in the ceiling-mounted camera, leading to no correspondences between the two views. Bottom: Using the proposed MSM, 41 correspondences (indicated by red crosses) are obtained between the two cameras in a fully automated manner, enabling the calibration of the two cameras

We provide additional quantitative results for evaluating the downstream task of body pose estimation. A 20-second multi-view video sequence was processed, from which 2D joint positions were extracted from 500 frames and triangulated using the DLT method to produce a 3D body pose. The same camera setup as in our real experiments (described in Section 3 of the main paper) was used. Since ground truth data for the 3D joints is unavailable, we rely on the reprojection error to evaluate the success rate of joint reconstruction. A joint is considered successfully reconstructed if its mean reprojection error falls below a specified distance threshold. The results, shown in Figure Fig. 7, compare joints visible in at least two camera views and those visible in at least three camera views, with the latter being less prone to overfitting. The ChArUco method and the proposed method produce nearly identical results.

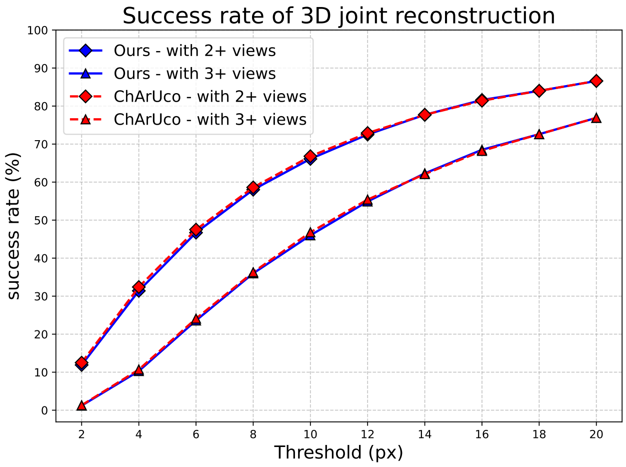

Figure 7 Success rates for 3D joint reconstruction at various thresholds. Comparison between the ChArUco method in red and our method in blue with joints either seen in at least 2 views or at least 3 views

References

[1] Romero-Ramirez, F.J., Muñoz-Salinas, R., Medina-Carnicer, R.: Speeded up detection of squared fiducial markers. Image and Vision Computing **76**, 38–47 (2018)

[2] Olson, E.: Apriltag: A robust and flexible multi-purpose fiducial system. Technical report, University of Michigan APRIL Laboratory (May 2010)

[3] Calvet, L., Gurdjos, P., Griwodz, C., Gasparini, S.: Detection and Accurate Localization of Circular Fiducials under Highly Challenging Conditions. In: Proceedings of the 2016 IEEE Conference on Computer Vision and Pattern Recognition (CVPR), Las Vegas, United States, pp. 562–570 (2016)

[4] Baker, S., Datta, A., Kanade, T.: Parameterizing homographies. Technical Report CMU-RI-TR-06-11, Carnegie Mellon University (March 2006)

[5] Moreno, D., Taubin, G.: Simple, accurate, and robust projector-camera calibration. In: 2012 Second International Conference on 3D Imaging, Modeling, Processing, Visualization & Transmission, pp. 464–471 (2012). IEEE
